# Treatment with liraglutide or naltrexone-bupropion in patients with genetic obesity: a real-world study

**DOI:** 10.1016/j.eclinm.2024.102709

**Published:** 2024-07-03

**Authors:** Mila S. Welling, Cornelis J. de Groot, Mostafa Mohseni, Renate E.H. Meeusen, Mariëtte R. Boon, Mieke M. van Haelst, Erica L.T. van den Akker, Elisabeth F.C. van Rossum

**Affiliations:** a Obesity Center CGG, Erasmus MC, University Medical Center Rotterdam, Rotterdam, the Netherlands; b Department of Internal Medicine, Division of Endocrinology, Erasmus MC, University Medical Center Rotterdam, Rotterdam, the Netherlands; c Division of Endocrinology, Department of Pediatrics, Erasmus MC-Sophia Children's Hospital, University Medical Center Rotterdam, Rotterdam, the Netherlands; d Department of Human Genetics, Amsterdam Reproduction and Development Research Institute, Amsterdam UMC, University of Amsterdam, Amsterdam, the Netherlands; e Emma Center for Personalized Medicine, Amsterdam UMC, University of Amsterdam, Amsterdam, the Netherlands; f Department of Pediatrics, IJsselland Hospital, Capelle aan den IJssel, the Netherlands

**Keywords:** Genetic obesity, Monogenetic obesity, Naltrexone-bupropion, Liraglutide, Pharmacotherapy

## Abstract

**Background:**

Rare genetic obesity commonly features early-onset obesity, hyperphagia, and therapy-resistance to lifestyle interventions. Pharmacotherapy is often required to treat hyperphagia and induce weight loss. We describe clinical outcomes of glucagon-like peptide-1 analogue liraglutide or naltrexone-bupropion treatment in adults with molecularly confirmed genetic obesity (MCGO) or highly suspected for genetic obesity without definite diagnosis (HSGO).

**Methods:**

We conducted a real-world cohort study at the Obesity Center CGG at Erasmus University Center, Rotterdam, Netherlands, between March 19, 2019, and August 14, 2023. All patients with MCGO and HSGO who were treated with either liraglutide or naltrexone-bupropion were included. Liraglutide 3 mg and naltrexone-bupropion were administered according to the manufacturer's protocol. Treatment evaluation occurred short-term, after 12 weeks on maximum or highest-tolerated dose, preceded by the 4–5 week dose escalation phase. Differences in anthropometrics, body composition, metabolic markers, self-reported appetite, eating behaviour, and quality of life (QoL) were evaluated.

**Findings:**

Ninety-eight adults were included in the analysis: 23 patients with MCGO and 75 patients with HSGO, with median BMI of 42.0 kg/m^2^ (IQR 38.7–48.2) and 43.7 kg/m^2^ (IQR 38.0–48.7), respectively. After liraglutide treatment, median weight at evaluation significantly decreased compared to baseline in both groups: −4.7% (IQR −6.0 to −1.5) in patients with MCGO and −5.2% (IQR −8.1 to −3.5) in patients with HSGO. Additionally, improvements were observed in appetite, fat mass, fasting glucose, and HbA1c in both patients with MCGO and with HSGO. Patients with HSGO also reported significant improvements in several domains of QoL and eating behaviour. In patients with MCGO and HSGO treated with naltrexone-bupropion, mean weight at evaluation significantly differed from baseline: −5.2% ± 5.8 in patients with MCGO and −4.4% ± 4.7 in patients with HSGO. Appetite, fat mass, and waist circumference significantly decreased in both groups. Obesity-related comorbidities improved in significant proportions of patients treated with liraglutide or naltrexone-bupropion.

**Interpretation:**

In conclusion, our short-term findings show potential of liraglutide and naltrexone-bupropion as treatment options for adults with (a clinical phenotype of) genetic obesity.

**Funding:**

MB, EvdA, and EvR are supported by the Elisabeth Foundation, a non-profit foundation supporting academic obesity research.

**Research in context**

**Evidence before this study**

We searched for articles written in English languages on PubMed, Embase, Medline, Web of Science Core Collection, Cochrane Central Register of Controlled Trials and Google Scholar databases from the onset of the databases until December 31, 2023. For this search, we used broad terms like: “genetic obesity”, “obesity”, “leptin-melanocortin pathway”, “pharmacotherapy”, “glucagon-like peptide-1”, “naltrexone-bupropion”, “naltrexone”, “bupropion”, “anti-obesity agents”, and “anti-obesity medication”. After this literature search, we concluded that literature on the conventional pharmacological treatments liraglutide and naltrexone-bupropion in patients with genetic obesity is scarce and only includes small sample sizes.

**Added value of this study**

In this study, we aimed to evaluate the effects of high-dosed liraglutide and naltrexone-bupropion in adults with a clinical phenotype of a genetic obesity disorder, including adults with molecularly confirmed genetic obesity and adults highly suspected for genetic obesity without definite diagnosis. We showed that treatment with liraglutide or naltrexone-bupropion, in addition to conventional lifestyle treatment, is associated with significant improvements in weight and appetite in a substantial portion of adults with (a clinical phenotype of) genetic obesity. Furthermore, we found significant improvements of various cardiometabolic measures, such as glycaemic indices and dyslipidaemia, various subscales of eating behaviour, and quality of life.

**Implications of all the available evidence**

We suggest that treatment with regular anti-obesity pharmacological agents, in addition to conventional lifestyle treatment, can be considered in patients with (a clinical phenotype of) genetic obesity, while closely monitoring treatment effects and potential adverse effects. Further research is needed to evaluate the long-term effects and investigate the effectiveness of certain anti-obesity medications for specific genetic obesity disorders.

## Introduction

Currently, the worldwide prevalence of overweight and obesity is approximately 39%.^1^ Obesity is a chronic relapsing disease, significantly increasing the risk of multiple other diseases such as type 2 diabetes, cardiovascular disease, cancer and depression.^2^ In recent years, there has been an increased emphasis on underlying causes of obesity. Although lifestyle factors as eating behaviour and physical exercise are very important factors involved in causing obesity, it is also known that other factors, such as weight-inducing medication, psychosocial factors, endocrine disease and (epi)genetic variation can contribute to the development of obesity as well.^3^ With the rapid development of technologies in genetic diagnostics in the last decade, the knowledge of underlying genetic causes of obesity has increased enormously.^4^ These genetic disorders are commonly divided into monogenic non-syndromic obesity (MO) and syndromic obesity (SO).^5^ More recently, the application of polygenic risk scores for obesity can further explain differences in inherited susceptibility to obesity.^6^ The common denominator in MO and SO diseases is that most of these patients have a defect in the leptin-melanocortin pathway that leads to early-onset obesity and hyperphagia (defined by impaired satiety and satiation leading to food-seeking behaviour).^4^ Examples of MO are melanocortin-4-receptor (MC4R), leptin-receptor (LEPR), and pro-opiomelanocortin (POMC) deficiency, while 16p11.2 deletion syndrome, Bardet-Biedl Syndrome (BBS) and deleterious mutations or methylation defects in *GNAS (*Guanine Nucleotide binding protein, Alpha Stimulating activity polypeptide) are often reported for SO.^4^^,^^7^^,^^8^ Such genetic defects as underlying cause of obesity was shown in a Dutch study in 9% of the 1230 patients visiting specialized obesity clinics, including definite and possible diagnosis.^9^

During multidisciplinary lifestyle interventions, patients with genetic obesity often respond insufficient in terms of weight loss as the hyperphagia remains untreated.^10^ Additionally, they are prone to regain weight if they succeed in losing weight.^11^ Therefore, additional pharmacotherapy, specifically centrally acting anti-obesity medication (AOM), is often necessary. In recent years, setmelanotide, a MC4R agonist, has been developed to stimulate the leptin-melanocortin pathway upstream of the defect that causes disruption in this pathway. In some genetic obesity disorders, it has been proven to effectively induce weight loss and improve quality of life.^12^^,^^13^ However, this drug is currently only available for a small fraction of patients with genetic obesity. Therefore, other AOMs, such as glucagon-like peptide-1 (GLP-1) analogues and naltrexone-bupropion, are also used to treat patients with genetic obesity. Liraglutide, initially developed for diabetes, was found to have weight reducing properties as it is known to both delay gastric emptying and beneficially influence the balance between orexigenic and anorexigenic neurons in the hypothalamus.^14^ Naltrexone-bupropion on the other hand, is a combination formulation acting on both the anorexic neurons of the hypothalamus, as well as on the dopamine driven reward system, thereby reducing food craving in both the homeostatic and the reward system.^15^ Literature on the results of these pharmacological treatments in patients with genetic obesity is, however, scarce and only includes small sample sizes.^16^, ^17^, ^18^

Given the need for data on pharmacological treatment in larger cohorts of patients with genetic obesity, we performed a real-world, observational study in which we will describe the results of treatment with liraglutide or naltrexone-bupropion. We will show these results in adults with a clinical phenotype of a genetic obesity disorder, including adults with molecularly confirmed genetic obesity (MCGO) and adults highly suspected for genetic obesity without definite diagnosis (HSGO). Our primary aim is to evaluate the effects on weight, while our secondary aim is to evaluate the effects on appetite and eating behaviour, body composition, obesity-related comorbidities, and quality of life.

## Methods

### Patients

All patients with MCGO and HSGO that were treated at Obesity Center CGG (Dutch: Centrum Gezond Gewicht; English: Center for Healthy Weight) with either liraglutide or naltrexone-bupropion between March 19, 2019, and August 14, 2023, were included in this study. The Obesity Center CGG at Erasmus University Center, Rotterdam, is together with the Clinical Genetics department at the Amsterdam UMC in Amsterdam, the (inter)national referral centre of expertise for genetic obesity disorders. MCGO was defined as a pathogenic (P) or likely pathogenic (LP) variant in a known genetic obesity gene according to the guidelines of the American College of Medical Genetics and Genomics (ACMG) for diseases with dominant inheritance (e.g. MC4R deficiency); two P or LP variants for recessive diseases (e.g. Bardet Biedl Syndrome and LEPR deficiency); or a loss of 16p11.2 region compatible with 16p11.2 deletion syndrome. We have opted to also include patients with HSGO in this study, since they exhibit a phenotype that is distinctly different from the common (often multifactorial) obesity and in our opinion also warrants a different approach in treatment. HSGO was defined as a combination of ≥2 features of genetic obesity, including early-onset of obesity, hyperphagia, presence of autism spectrum disorder and/or intellectual deficit, striking weight differences with family members, congenital abnormalities suggestive of a genetic obesity disorder (e.g. rod-cone dystrophy in Bardet-Biedl Syndrome), or specific dysmorphic features.^5^ This group also included patients of whom genetic test results revealed variants of unknown significance (VUS) in obesity-related genes.

The standard procedure for all patients visiting our obesity centre is that at first visit a diagnostic work-up is performed, including obtaining clinical patient characteristics, such as self-reported age-of-onset of obesity, appetite characteristics and medical history, using a comprehensive standardised questionnaire that is extensively discussed at the first visit.

### Ethics

This study was approved by the local ethics committee (MEC-2023-0029) and conducted according to the principles of the declaration of Helsinki. For patients visiting our obesity centre before April 2022, the need for informed consent was waived by the local ethics committee, after this date, all patients provided informed consent.

### Pharmacological treatment

After the initial evaluation and diagnostic work-up, a personalized treatment plan is made for each patient considering their individual needs regarding adjustment of eating, exercise, and sleeping behaviour, and psychological support. Pharmacological treatment is considered as add-on therapy in patients with a history of therapy resistance to conventional lifestyle treatment or weight regain after initial weight loss. The choice for either liraglutide or naltrexone-bupropion was made in a shared-decision making process taking into account the personal preferences of the patient, possible side effects and contra-indications, presence of co-morbidities, specific eating behaviour, and whether pharmacotherapy was reimbursed by the patient's insurance company. Pharmacotherapy is initiated according to the manufacturer's recommended protocol. In four to five weeks, naltrexone-bupropion is escalated to a maximum dose of 32/360 mg per day and liraglutide to 3.0 mg/day, or to the highest tolerated dose if maximum dose could not be reached due to side effects. A follow-up visit is planned 12 weeks after the maximum or highest tolerated dose is reached to assess and evaluate the effect of pharmacotherapy. This total treatment duration of approximately 16 weeks was chosen as the weight loss achieved in this period is useful in identifying patients who would experience additional clinically meaningful weight loss, as is also recommended in international guidelines.^19^, ^20^, ^21^ When treatment with the initial AOM was discontinued due to e.g. significant side effects or lack of significant effect, treatment with the other available AOM was offered. Patients can, therefore, be included in the analysis multiple times (maximum three times). In this study, no patients were treated with both liraglutide and naltrexone-bupropion simultaneously. Baseline measurements of anthropometrics, laboratory measurements and questionnaires were performed before each pharmacotherapeutical treatment, also in case of multiple pharmacological treatments in one patient.

### Clinical assessment

At first visit, ethnicity was determined using birth country of parents. Level of education was categorized in three groups: 1) low (i.e., no education, (special) primary education, lower secondary or vocational education); 2) middle (i.e., upper secondary education, intermediate vocational education); 3) high (i.e., higher vocational education or university education). During the first and follow up visit, height, weight, waist circumference, blood pressure, and body composition were measured. Height was measured using a wall-mounted, calibrated stadiometer. Weight was measured with the patient clothed except from their shoes using a calibrated scale. Body mass index (BMI, in kg/m^2^) was calculated as weight in kilograms divided by height in meters squared. Blood pressure was measured using an automatic blood pressure monitor (DinaMap Monitor; GE Health Care, Freiburg, Germany). Body composition was measured using bio-impedance analysis (BIA, Inbody S10, BioSpace, Seoul, Korea). Self-reported appetite was clinically assessed at both the first visit and follow-up visit using semi-structured interviewing techniques that explored various domains of appetite, including feelings of hunger, satiety, and satiation. Examples of questions included: “Can you describe your appetite?”, “Is your appetite less than others, as appropriate, or bigger than others?”, ‘‘Do you feel satiated after a normal sized meal?”, and “How many hours does this feeling of satiation last?”. Additionally, we explicitly asked patients at the follow-up visit whether they had experienced any changes in appetite. Improvement in appetite was defined as reporting an increased satiety and/or increased satiation. Side effects were assessed by the treating physician via a telephone consult at 5 weeks of treatment and at the follow-up visit.

### Laboratory tests

At baseline and at follow-up, a fasted blood sample was drawn. Metabolic parameters were measured according to standard procedures, including glucose, insulin, HbA1c, total cholesterol, high-density lipoprotein (HDL) cholesterol, low-density lipoprotein (LDL) cholesterol, triglyceride, aspartate aminotransferase (AST), alanine aminotransferase (ALT), gamma-glutamyltransferase (GGT), and alkaline phosphatase (AP).

Presence of comorbidities was defined using the following criteria:

- Type 2 diabetes using the ADA criteria: HbA1c ≥ 48 mmol/mol, a fasting glucose ≥7.0 mmol/l, a random glucose level of ≥11.1 mmol/l, or current use of antidiabetic medication.^22^
- Dyslipidaemia as any elevation in total cholesterol, LDL-cholesterol or triglycerides or lowered levels of HDL-cholesterol, using the cut-offs described by Balder.^23^
- Elevated liver enzymes as any level of ALT, AST, GGT or AP above the reference range as described by Schumann.^24^
- Metabolic syndrome using the criteria of the joint interim statement by Alberti et al.^25^

Resolution of a comorbidity was defined as all parameters, included in the definition of that comorbidity, were within normal range.

### Questionnaires

Eating behaviour was assessed using two questionnaires:

- The Dutch Eating Behaviour Questionnaire (DEBQ) assesses 3 eating behaviours, namely restrained eating (tendency to eat less to lose body weight), emotional eating (eating in response to emotional events), and external eating (eating triggered by the presence of external food cues).^26^ Higher scores reflect a stronger tendency towards the respective eating behaviour.
- The Eating Disorder Examination Questionnaire (EDE-Q) version 5 explores 4 domains, namely restraint, eating concern, shape concern and weight concern.^27^ Higher scores reflect more severe symptoms of eating behaviour associated with eating disorders.

Quality of life (QoL) was assessed using two questionnaires:

- General QoL was evaluated using the EuroQol Five Dimensions Health Questionnaire (EQ-5D).^28^ Scores can range between −0.446 and 1.0, with higher scores reflecting higher QoL.
- Obesity-related QoL was assessed using the OBESI-Q, which assesses QoL in six domains (eating behaviour, social functioning, psychological functioning, physical function, body image, and sexual functioning). This questionnaire entails a set of six validated subscales of de BODY-Q.^29^ Scores range between 0 and 100, with higher scores reflecting a better obesity-related QoL.

Most of the patients included in this study with intellectual disability or autism spectrum disorder were able to complete the questionnaires themselves. In case of problems filling out the questionnaires, caregivers were asked to help the patient.

### Statistical analysis

All statistical analyses were done using IBM SPSS Statistics 28.0. Depending on data distribution, data are shown as mean ± standard deviation or median [interquartile range (IQR)]. When patients were included twice or thrice, only baseline characteristics depicted in Table 1 at start of first pharmacotherapy were used. Differences in treatment outcomes measured at start of treatment and at the follow-up visit were tested using either paired t-tests or Wilcoxon signed rank tests. Furthermore, we compared treatment outcomes between the two included groups using independent t-tests, Mann–Whitney U tests and χ2 tests/Fisher's exact tests, as appropriate. The effect of weight loss on resolving obesity-related comorbidities was investigated by stratifying patients into two groups: those with ≤5% weight loss and those with ≥5% weight loss. A per-protocol analysis was conducted for both primary and secondary outcomes, including only patients who completed the 16 weeks of treatment. The primary outcome was compared between patients with and without bariatric surgery and between females and males. An intention-to-treat analysis was conducted for the primary outcome as well. Lastly, sensitivity analyses on the primary outcome were performed including only treatment outcomes of initial pharmacological treatments, while excluding outcomes of subsequent consecutive treatments. The 95% confidence intervals (CI) are reported for all outcomes. These 95% CI convey information about the size and precision of the observed effect, determined by the size of the study sample.^30^^,^^31^ In addition, we calculated post-hoc the power on the difference between baseline and after 16 weeks of treatment on the main outcome of percentage weight loss for liraglutide and naltrexone-bupropion in both the MCGO and HSGO group with a two-sided α of 0.05.

**Table 1:** Baseline characteristics in all subgroups.

	Genetic obesity (*n* = 23)^a^	High suspicion of genetic obesity (*n* = 75)^b^
**Female, n (%)**	14 (60.9)	58 (77.3)
**Age at intake, year †**	23.0 (20.0–31.0)	34.0 (24.0–44.0)
**Ethnicity, n (%)**		
Dutch	18 (78.3)	56 (77.8)
Western	2 (8.7)	7 (9.7)
Non-Western	3 (13.0)	9 (12.5)
**Education level, n (%) †**		
Low	12 (52.2)	10 (13.5)
Middle	7 (30.4)	23 (31.1)
High	4 (17.4)	41 (55.4)
**Age of onset of obesity, year**	6.0 (2.0–13.0)	6.0 (3.0–12.0)
**Increased appetite, n (%) †**	19 (82.6)	30 (40.5)
**History of bariatric surgery, n (%)**	2 (8.7)	13 (17.3)
*Anthropometrics and vital functions*		
**Weight, kg**	128.5 ± 30.1	133.0 ± 32.2
**BMI, kg/m**^**2**^	42.0 (38.7–48.2)	43.7 (38.0–48.7)
**Obesity class, n (%)**		
Overweight	2 (8.7)	1 (1.4)
I	2 (8.7)	11 (14.9)
II	4 (17.4)	14 (18.9)
III	15 (65.2)	48 (64.9)
**Waist circumference, cm**	127.7 ± 23.2	126.2 ± 19.4
**SBP, hg/mm**	127 (122–156)	137 (126–152)
**DBP, hg/mm**	80 ± 13	83 ± 14
**Body composition, kg**		
Fat mass	61.0 (50.9–73.5)	63.1 (47.8–77.5)
Fat-free mass	68.8 (53.5–75.8)	67.0 (58.7–74.8)
*Co-morbidities*		
**Type 2 diabetes, n (%)**	1 (4.3)	9 (12.0)
**Dyslipidaemia, n (%**)	12 (54.5)	24 (32.4)
**Elevated liver enzymes, n (%)**	14 (66.7)	32 (43.2)
**Metabolic syndrome, n (%)**	13 (72.2)	44 (69.8)
*Questionnaires*		
**EDE-Q total score**	2.3 ± 1.1	3.0 ± 1.3
**DEBQ**		
Restrained ‡	2.9 (2.6–3.4)	3.6 (3.1–4.1)
Emotional	2.7 (1.8–3.1)	2.6 (1.5–3.4)
External	2.9 ± 0.6	2.7 ± 0.9
**EQ (5D) total score ‡**	0.85 (0.77–0.93)	0.67 (0.61–0.82)
**OBESI-Q**		
Psychological	57.8 ± 17.2	50.5 ± 19.7
Social	52.0 (43.5–62.8)	52.0 (40.0–68.0)
Eating behaviour	52.4 ± 15.5	55.6 ± 14.3
Body image	30.8 ± 20.6	21.5 ± 19.2
Physical	74.4 ± 11.3	65.6 ± 19.6
Sexual	49.3 ± 16.3	48.6 ± 22.4
*Intervention*		
**Use of AOM, n (%)**		
Liraglutide	18 (62.1)	60 (73.2)
Naltrexone-bupropion	11 (37.9)	22 (26.8)

Abbreviations: BMI, body mass index; SBP, systolic blood pressure; DBP, diastolic blood pressure; EDE-Q, Eating Disorder Examination Questionnaire; DEBC, Dutch Eating Behaviour Questionnaire; EQ-5D, EuroQol 5D; AOM, anti-obesity medication.

Data is displayed as mean ± standard deviation or median (IQR) and only shown for patients once. Significant different between patients with genetic obesity and patients with a high suspicion of genetic obesity: †p ≤ 0.001; ‡p < 0.01.

a Data available for waist circumference in n = 14, SBP and DBP in n = 15, fat mass in n = 21, dyslipidaemia in n = 22, elevated liver enzymes in n = 21, metabolic syndrome in n = 18, EDE-Q in n = 11, DEBC and OBESI-Q social/body image/physical domain in n = 18, EQ (5D) and OBESI-Q psychological domain in n = 17, and OBESI-Q eating behaviour domain in n = 11 and OBESI-Q sexual domain in n = 6.

b Data available for ethnicity in n = 72, education level, increased appetite, weight, BMI, dyslipidaemia, and elevated liver enzymes in n = 74, age of onset of obesity in n = 71, waist circumference in n = 57, SBP, and DBP in n = 58, fat mass, DEBQ, EQ-5D, and OBESI-Q body image and physical domain in n = 60, metabolic syndrome in n = 63, EDE-Q in n = 48, OBESI-Q psychological and social domain in n = 59, OBESI-Q eating behaviour domain in n = 45, and OBESI-Q sexual domain in n = 39.

### Role of funding sources

The funder of the study had no role in study design, data collection, data analysis, data interpretation, or writing of the report.

## Results

### Baseline characteristics

Baseline characteristics are summarized in Table 1. A total of 98 unique patients with a clinical picture of genetic obesity were included, of which *n =* 23 with molecularly confirmed genetic obesity (MCGO) and *n =* 75 with a high suspicion of genetic obesity (HSGO). The genetic diagnoses of patients with MCGO are summarized in Table S1. In 74 out of 75 patients with HSGO, genetic analysis was performed. These results revealed a VUS in an obesity-related gene in 18 patients (Table S2). In 29 of 75 patients, additional genetic analyses, such as array analysis, methylation analysis, or whole exome sequencing, was performed by clinical geneticists. In none of the patients did this reveal a pathogenic genetic defect related to their obesity. These 98 patients together completed 111 pharmacological treatments. Thirteen patients had separate pharmacotherapeutical treatments consecutively: four patients with MCGO and five patients with HSGO had two consecutive treatments and one patient with MCGO and one patient with HSGO had three consecutive treatments. In total, 78 patients were treated with liraglutide, and 33 patients were treated with naltrexone-bupropion. One patient was treated with bupropion monotherapy. Reasons for not completing this treatment phase, including the proportion of patients who discontinued treatment because of side-effects, during liraglutide (*n =* 10) or naltrexone-bupropion (*n* = 9) are summarized in Table S3.

### Change in weight after pharmacological treatment

**Fig. 1:**
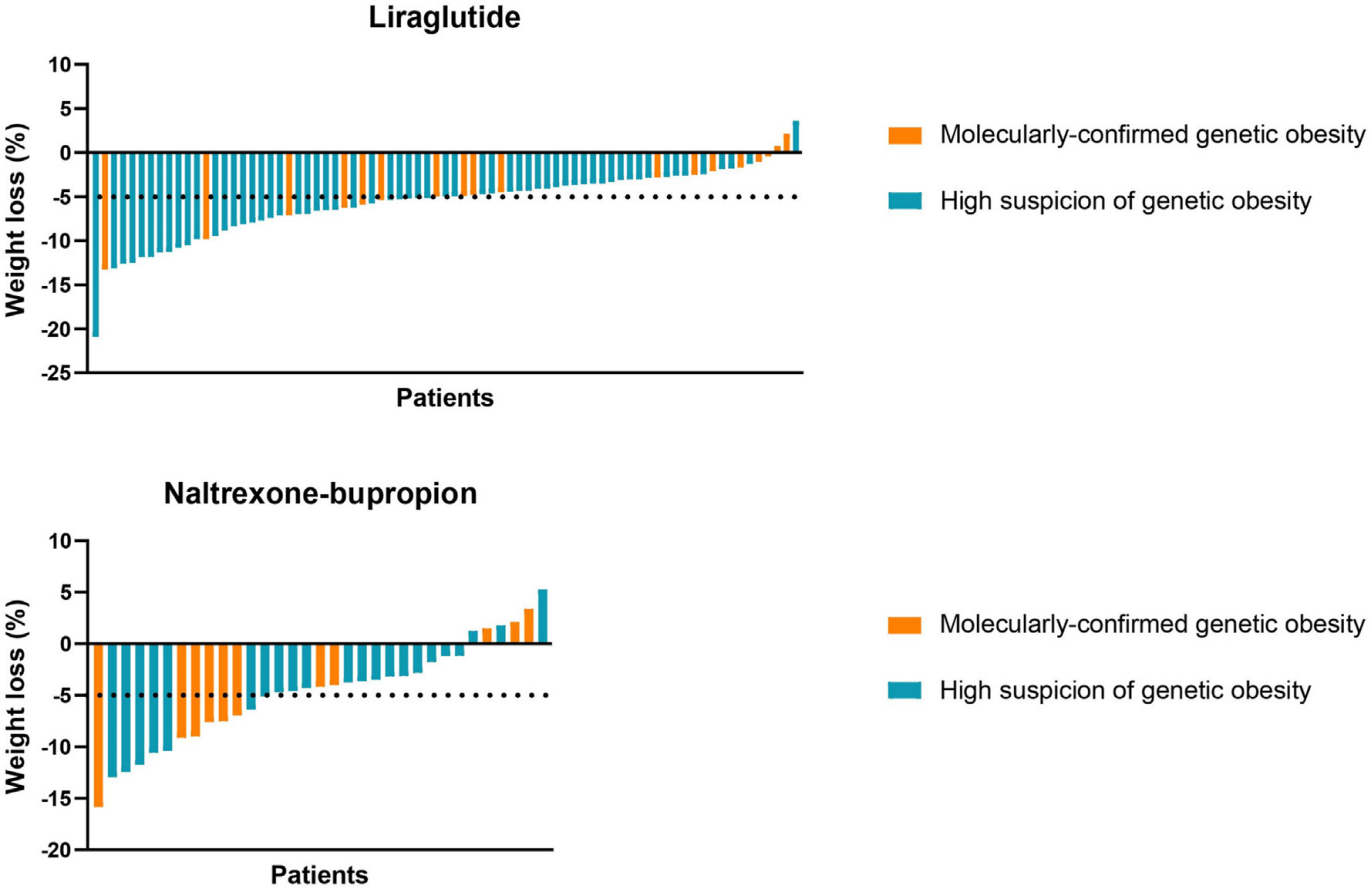
**Percentage of weight loss depicted for liraglutide and naltrexone-bupropion separately**.

**Fig. 2:**
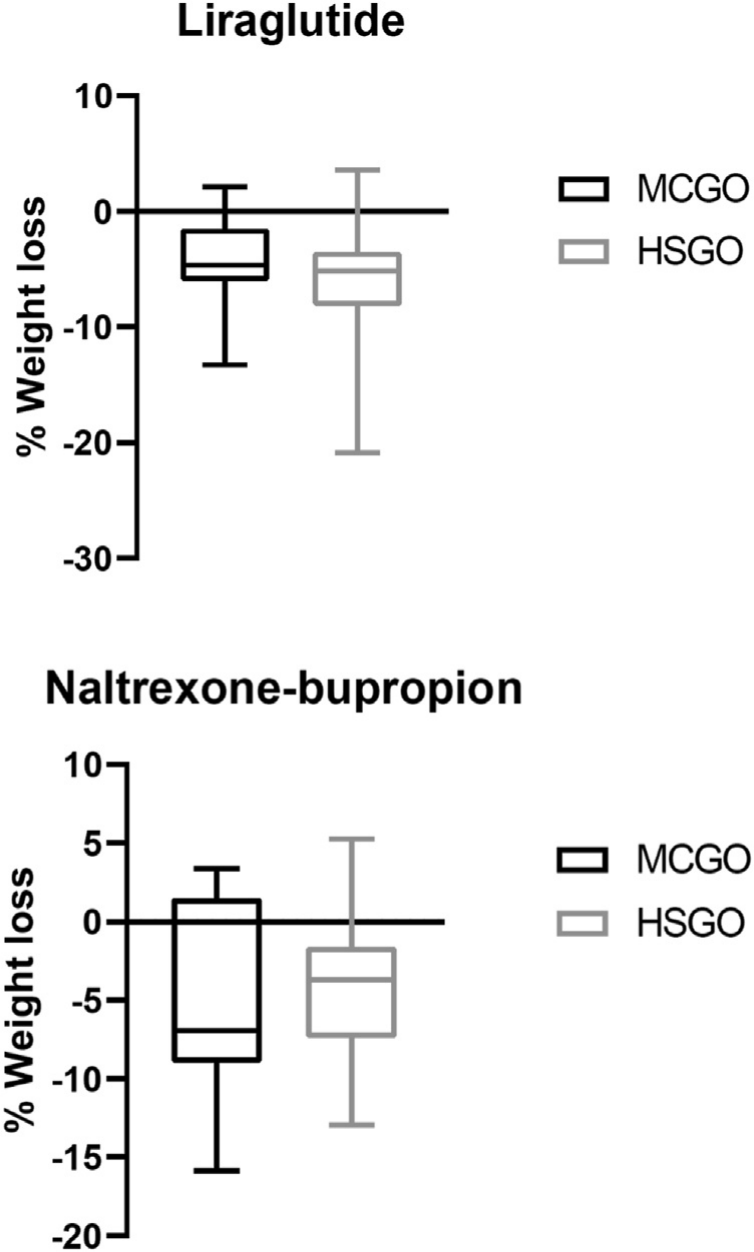
**Percentage of weight loss depicted for molecularlyconfirmed genetic obesity (MCGO) and high suspicion of genetic obesity (HSGO) after 16 weeks of liraglutide or naltrexone-bupropion treatment**. Abbreviations: MCGO, adults with molecularly confirmed genetic obesity; HSGO, adults highly suspected for genetic obesity without definite diagnosis.

The effects on weight loss are summarised in Table 2 and Figs. 1 and 2.

**Table 2:** Weight loss during treatment with liraglutide or naltrexone-bupropion treatment in both subgroups.

	Genetic obesity (n = 29)^a^	p-value	High suspicion of genetic obesity (n = 82)^b^	p-value
*Liraglutide*	*n* = 18		*n* = 59^c^	
**Δ Weight,**				
Kg	−5.4 (−8.5 to −2.1)	**<0.001**	−7.2 (−10.5 to −4.5)	**<0.001**
%	−4.7 (−6.0 to −1.5)		−5.2 (−8.1 to −3.5)	
**Change in weight, categorized, n (%)**^d^				
Weight gain	2 (11.1)		1 (1.7)	
0–5% weight loss	10 (55.6)		27 (45.8)	
5–10% weight loss	5 (27.8)		22 (37.3)	
10–20% weight loss	1 (5.6)		8 (13.6)	
>20% weight loss	0		1 (1.7)	
Highest weight loss, %	−13.3%		−20.9%	
*Naltrexone-bupropion*	*n* = 11		*n* = 22	
**Δ Weight**,				
Kg	−6.6 ± 9.4	**0.044**	−5.2 ± 6.0	**<0.001**
%	−5.2 ± 5.8		−4.4 ± 4.7	
**Change in weight, categorized, n (%)**^d^				
Weight gain	3 (27.3)		3 (13.6)	
0–5% weight loss	2 (18.2)		12 (54.5)	
5–10% weight loss	5 (45.5)		2 (9.1)	
10–20% weight loss	1 (9.1)		5 (22.7)	
Highest weight loss, %	−15.8%		−13.0%	

Abbreviations: kg, kilogram; %, percentage.

Data is displayed as mean ± standard deviation or median (IQR). Significant p-values are presented in bold when p < 0.05.

a Four patients with genetic obesity had two separate consecutive treatments, one patient with genetic obesity had three separate consecutive treatments.

b Five patients with a high suspicion of genetic obesity had two separate consecutive treatments, one patient with a high suspicion of genetic obesity had three separate consecutive treatments.

c In one patient with a high suspicion of genetic obesity no anthropometrics were available at evaluation moment.

d 0–5% is defined as 0–4.99%, 5–10% is defined as 5–9.99%, 10–15% is defined as 10–14.99%, 15–20% is defined as 15–19.99%.

After liraglutide treatment, median weight was significantly lower than at baseline in both patients with MCGO and HSGO (Fig. 2). Weight loss after liraglutide treatment was −5.4 kg (IQR −8.5 to −2.1, 95% CI −7.4 to −3.1), corresponding with −4.7% (IQR −6.0 to −1.5, 95% CI −6.0 to −2.3), for patients with MCGO. For patients with HSGO, weight loss was −7.2 kg (IQR −11.1 to −4.5, 95% CI −9.3 to −6.5), corresponding with −5.2% (IQR −8.1 to −3.5, 95% CI −7.1 to −5.1), after 16 weeks of liraglutide treatment. After treatment with naltrexone-bupropion, mean weight significantly decreased in both groups. Patients with MCGO lost on average −6.6 kg ± 9.4 (95% CI −12.9 to −0.2) of body weight, corresponding with −5.2% ± 5.8 (95% CI −9.1 to −1.3). A mean decrease in body weight of −5.2 kg ± 6.0 (95% CI −7.9 to −2.6), corresponding with −4.4% ± 4.7 (95% CI −6.6 to −2.4), was observed in patients with HSGO.

Of the patients with MCGO, 33.4% of the patients treated with liraglutide and 54.6% of patients treated with naltrexone-bupropion reached a weight loss of ≥5%. For the patients with HSGO, this was 52.6% and 31.8%.

Sensitivity analyses showed no evident differences in weight loss between the total cohort, including treatment outcomes of both initial and subsequent consecutive treatments, and the group, including treatment outcomes of only initial treatments. Cases of all patients with multiple consecutive treatments are described in Table S4.

Nine patients, of which three patients treated with liraglutide and six patients treated with naltrexone-bupropion, did not lose weight during treatment. Three out of the six patients who did not lose weight (ranging between +1.5% and +3.4%) during naltrexone-bupropion treatment were patients with MCGO, all diagnosed with proximal 16p11.2 deletion syndrome. There were, however, two patients with proximal 16p11.2 deletion syndrome who were successfully treated with naltrexone-bupropion (weight loss of −4.2% and −9.0%). In the liraglutide treated group, there was one patient with MCGO with a heterozygous MC4R variant who did not lose weight (+0.7%). In contrast, six other patients with heterozygous MC4R variants were able to lose weight (ranging between −2.1 and −13.3%).

For both liraglutide and naltrexone-bupropion treatment, no statistically significant differences in percentage weight loss were observed between patients with and without a history of bariatric surgery. Additionally, sex did not seem to have strong effect on this outcome for both agents.

Post-hoc power analysis showed a power for the difference in percentage weight loss between baseline and after 16 weeks of treatment with liraglutide for MCGO of 0.96 and HSGO of >0.99. For naltrexone-bupropion, the power for MCGO was 0.77 and for HSGO of 0.97.

### Other clinical outcomes after pharmacological treatment

The results of the analysis of secondary outcome measures are summarised in Table 3 for liraglutide and Table 4 for naltrexone-bupropion.

**Table 3:** Differences in secondary endpoints between start of treatment and evaluation moment of liraglutide treatment.

	Genetic obesity (*n* = 18)^c^	95% CI	p-value	High suspicion of genetic obesity (*n* = 60)^d^	95% CI	p-value
**Treatment duration, months**	4.0 (3.6–4.3)			4.2 (3.8–5.1)		
**Δ WC**,						
Cm	−2.8 ± 4.5	−6.5 to 1.0	0.125	−3.4 ± 7.4	−5.9 to −0.9	**0.009**
%	−2.2 ± 3.4	−5.0 to 0.7		−2.6 ± 5.8	−4.5 to −0.7	
**Δ SBP, mm/Hg**	−2 ± 17	−14 to 10	0.693	−6 ± 14	−11 to −2	**0.006**
**Δ DBP, mm/Hg**	0 ± 10	−7 to 7	0.900	−1 ± 10	−4 to 3	0.682
**Δ Body composition**,						
Fat mass, kg	−3.3 ± 6.1	−7.7 to 1.0	0.119	−4.8 ± 6.0	−7.1 to −2.5	**<0.001**
Fat mass, %	−0.9 ± 2.7	−2.8 to 1.0	0.334	−1.0 ± 3.0	−2.1 to 0.2	**0.098**
Fat-free mass, kg	−0.7 ± 3.0	−2.8 to 1.5	0.509	−3.1 ± 2.5	−4.0 to −2.1	**<0.001**
**Δ Fasting glucose, mmol/l**	−0.2 (−0.8–0)	−0.6 to 0.01	**0.035**	−0.3 (−0.6 to −0.1)	−0.5 to −0.2	**<0.001**
**Δ HbA1c, mmol/mol**	−1.5 (−4.0 to −0.3)^e^	−3.8 to −0.7	**0.007**	−3.0 (−4.0 to −2.0)^f^	−4.8 to −2.8	**<0.001**
**Δ Fasting insulin, pmol/l**	0 (−34 to 107)	−46 to 105	0.400	−14 (−48 to 25)	−22 to 19	0.159
**Dyslipidaemia, n (%)**^b^						
Still present	9 (90.0)			13 (76.5)		
Improved	1 (10.0)			4 (23.5)		
**Elevated liver enzymes, n (%)**^a^						
Still present	8 (72.7)			15 (75.0)		
Improved	3 (27.3)			5 (25.0)		
**Metabolic syndrome, n (%)**^a^						
Still present	7 (87.5)			24 (80.0)		
Improved	1 (5.6)			6 (20.0)		
**Improved appetite, n (%)**^a^	15 (83.3)			47 (87.0)		
**Δ EDE-Q total score**	−0.5 (−1.2 to 0.2)	−1.2 to 0.2	0.173	−0.4 (−0.8–0.1)	−0.8 to −0.2	**0.001**
**Δ DEBQ**						
Emotional	−0.08 (−0.58 to 0.04)	−0.51 to 0.04	0.089	−0.15 (−0.54 to 0.15)	−0.45 to −0.03	**0.027**
Restrained	0.18 ± 0.45	−0.11 to 0.47	0.201	−0.13 ± 0.59	−0.31 to 0.06	0.175
External	0.04 ± 1.05	−0.62 to 0.71	0.307	−0.04 ± 0.75	−0.28 to 0.19	0.088
**Δ EQ-5D total score**	0.00 (0.00–0.09)	−0.03 to 0.12	0.225	0.02 (0.00–0.11)	0.004 to 0.09	**0.035**
**Δ OBESI-Q**						
Eating behaviour	7.8 ± 15.5	−8.4 to 24.1	0.270	3.6 ± 17.0	−3.0 to 10.2	0.277
Social	5.5 (−6.5 to 17.5)	−1.9 to 14.4	0.155	2.0 (−7.0 to 8.0)	−4.5 to 4.1	0.561
Psychological	3.2 ± 19.1	−9.6 to 16.0	0.593	3.8 ± 14.9	−0.9 to 8.5	0.111
Physical	6.0 (0.0–14.0)	−21.0 to 20.0	0.109	4.5 (−6.5 to 11.8)	−4.7 to 9.1	0.098
Body image	12.3 ± 19.1	0.2 to 24.2	**0.046**	8.3 ± 17.6	2.8 to 13.8	**0.004**
Sexual	4.0 and 7.5	−37.0 to 52.0	–	5.6 ± 14.8	−0.2 to 11.5	0.058

Abbreviations; Δ, delta; WC, waist circumference; SBP, systolic blood pressure; DBP, diastolic blood pressure; HbA1c, haemoglobin A1c; EDE-Q, Eating Disorder Examination Questionnaire; DEBQ, Dutch Eating Behaviour Questionnaire; EQ 5D, EuroQol 5D; 95% CI, 95% confidence interval.

Data is displayed as mean ± standard deviation or median (IQR). Significant p-values are presented in bold when p < 0.05.

a Appetite was self-reported assessed at first and follow-up visit.

b Data only shown for patients who had the obesity-related comorbidity at baseline.

c Data available for waist circumference in n = 8, SBP, DBP, and body composition in n = 10, glucose and HbA1c in n = 16, insulin in n = 7, dyslipidaemia in n = 10, elevated liver enzymes in n = 11, metabolic syndrome in n = 18, EDE-Q in n = 6, EQ-5D in n = 11, DEBQ, OBESI-Q social, body image, and physical activity domain in n = 12, OBESI-Q psychological domain in n = 11, OBESI-Q eating behaviour domain in n = 6, OBESI-Q sexual domain in n = 15.

d Data available for appetite in n = 54, waist circumference in n = 37, SBP and DBP in n = 39, body composition in n = 29, fasting glucose in n = 50, HbA1c in n = 52, and fasting insulin in n = 44, dyslipidaemia in n = 17, elevated liver enzymes in n = 20, metabolic syndrome in n = 30, EDE-Q in n = 15, EQ-5D in n = 22, DEBQ, OBESI-Q body image and physical activity in n = 12, OBESI-Q psychological domain in n = 11, OBESI-Q social domain in n = 11, OBESI-Q eating behaviour domain in n = 6, OBESI-Q sexual domain in n = 15.

e Median HbA1c at baseline was 35 mmol/mol (IQR 34–38).

f Median HbA1c at baseline was 37 mmol/mol (IQR 35–41).

**Table 4:** Differences in secondary endpoints between start of treatment and evaluation moment of naltrexone-bupropion treatment.

	Genetic obesity (*n* = 11)^c^	95% CI	p-value	High suspicion of genetic obesity (*n* = 22)^d^	95% CI	p-value
**Treatment duration, months**	4.5 (4.0–5.7)			4.9 (4.5–5.6)		
**Δ WC,**						
Cm	−6.6 ± 3.2	−10.6 to −2.7	**0.010**	−7.0 ± 4.6	−10.3 to −3.7	**<0.001**
%	−5.5 ± 2.6	−8.7 to −2.2		−5.4 ± 3.3	−7.8 to −3.0	
**Δ SBP, mm/Hg**	0 (−3 to 33)	−15 to 39	0.285	−3.5 (−7 to 5)	−21 to 6	0.410
**Δ DBP, mm/Hg**	−1 ± 9	−13 to 10	0.756	0 ± 17	−10 to 11	0.946
**Δ Body composition**,						
Fat mass, kg	−6.6 (−8.3 to −5.1)	−17.1 to −0.6	**0.028**	−3.9 (−10.8 to −1.5)	−10.0 to 1.4	**0.005**
Fat mass, %	−3.9 ± 2.8	−6.5 to −1.3	**0.010**	−1.6 ± 2.1	−3.2 to −0.1	**0.036**
Fat-free mass, kg	−0.1 ± 2.8	−2.7 to 2.5	0.939	−2.1 ± 2.2	−3.7 to −0.5	**0.014**
*Laboratory parameters*						
**Δ Fasting glucose, mmol/l**	0.2 (−0.2 to 0.5)	−0.2 to 0.4	0.264	0.0 (−0.3 to 0.4)	−0.6 to 0.5	0.657
**Δ HbA1, mmol/mol**	−0.5 (−2.5 to 1.0)^e^	−2.6 to 0.8	0.260	−1.0 (−2.5 to −0.0)^f^	−3.4 to 0.6	0.057
**Δ Fasting insulin, pmol/l**	−25 (−46 to 34)	−50 to 48	0.575	−12 (−38 to 5)	−50 to 18	0.164
**Dyslipidaemia, n (%)**^a^						
Still present	5 (71.4)			6 (75.0)		
Improved	2 (28.6)			2 (25.0)		
**Elevated liver enzymes, n (%)**^a^						
Still present	3 (50.0)			6 (54.5)		
Improved	3 (50.0)			5 (45.5)		
**Metabolic syndrome, n (%)**^b^						
Still present	3 (75.0)			9 (100.0)		
Improved	1 (25.0)			0		
**Improved appetite, n (%)**^a^	10 (90.9)			17 (81.0)		
**Δ EDE-Q total score**	−0.07 ± 1.4	−1.78 to 1.65	0.920	−0.14 ± 0.66	−0.56 to 0.28	0.471
**Δ DEBQ**						
Restrained	−0.08 ± 0.49	−0.59 to 0.43	0.693	0.05 ± 0.32	−0.15 to 0.25	0.596
Emotional	−0.40 ± 0.72	−1.15 to 0.36	0.233	−0.24 ± 0.53	−0.58 to 0.09	0.137
External	−0.04 ± 0.46	−0.52 to 0.44	0.394	−0.06 ± 0.81	−0.57 to 0.46	0.555
**Δ EQ-5D total score**	0.09 (−0.06 to 0.15)	−0.06 to 0.17	0.173	0.06 (0.00–0.14)	−0.09 to 0.16	0.093
**Δ OBESI-Q**						
Eating behaviour	6.8 ± 6.7	−0.2 to 13.8	0.054	2.7 ± 7.8	−2.7 to 7.6	0.260
Social	9.8 ± 25.5	−16.9 to 36.6	0.388	−3.0 ± 10.3	−9.6 to 3.6	0.335
Psychological	4.7 ± 18.5	−14.7 to 24.1	0.563	−0.6 ± 13.3	−9.0 to 7.9	0.882
Physical	8.5 ± 8.6	−0.5 to 17.5	0.059	−1.7 ± 9.7	−7.8 to 4.5	0.563
Body image	12.3 ± 13.0	−1.3 to 26.0	0.068	1.5 ± 8.7	−4.0 to 7.0	0.562
Sexual	N.A.			3.2 ± 10.1	−7.4 to 13.8	0.477

Abbreviations; Δ, delta; WC, waist circumference; SBP, systolic blood pressure; DBP, diastolic blood pressure; HbA1c, haemoglobin A1c; EDE-Q, Eating Disorder Examination Questionnaire; DEBQ, Dutch Eating Behaviour Questionnaire; EQ5D, EuroQol 5D; N.A., not available; 95% CI, 95% confidence interval.

Data is displayed as mean ± standard deviation or median (IQR). Significant p-values are presented in bold when p < 0.05.

a Appetite was self-reported assessed at first and follow-up visit.

b Data only shown for patients who had the obesity-related comorbidity at baseline.

c Data is available for WC, SDP, DBP, and EDE-Q in n = 5, body composition in n = 7, HbA1c, insulin in n = 10, elevated liver enzymes in n = 6, dyslipidaemia in n = 7, and metabolic syndrome n = 4, EQ-5D, DEBQ and OBESI-Q psychological, social, eating behaviour, body image and physical activity domain in n = 6, OBESI-Q sexual domain in n = 0.

d Data is available for appetite in n = 21, WC and body composition in n = 10, SBP, DBP, EDE-Q, EQ-5D, DEBC, and OBESI-Q psychological, social, eating behaviour, body image and physical activity domain in n = 12, HbA1c, fasting glucose and insulin in n = 21, dyslipidaemia in n = 8. Elevated liver enzymes in n = 12, metabolic syndrome in n = 9, OBESI-Q sexual domain in n = 6.

e Median HbA1c at baseline was 34 mmol/mol (IQR 32–35).

f Median HbA1c at baseline was 36.5 mmol/mol (IQR 34–40).

#### Appetite and eating behaviour

In patients treated with liraglutide, self-reported appetite decreased in 83.3% of patients with MCGO and in 87.0% of patients with HSGO. Signs of eating behaviours associated with eating disorders decreased significantly with −0.4 points in patients with HSGO (p = 0.001). Based on DEBQ, there was a significant improvement of −0.15 (IQR −0.54 to −0.15, p = 0.027) in emotional eating in patients with HSGO.

While naltrexone-bupropion treatment, improvements in self-reported appetite was reported in 90.9% of patients with MCGO and 81.0% of patients with HSGO. Additionally, trends toward significant improvements in EDE-Q and most domains of eating behaviours, assessed using the DEBQ, were observed in both groups.

#### Body composition

In patients with HSGO who were treated with liraglutide, waist circumference decreased (−3.4 cm ± 7.4, p = 0.009) significantly compared to baseline. Additionally, absolute fat mass (−4.7 kg ± 5.8, p < 0.001), but also in a smaller amount fat-free mass (−3.1 kg ± 2.5, p < 0.001), decreased significantly in these patients. These differences were not observed in the patients with MCGO.

In patients treated with naltrexone-bupropion, significant reductions in waist circumference (−6.6 cm ± 3.2 and −7.0 cm ± 4.6, p = 0.010 and p < 0.001, resp.), absolute fat mass (−6.6 kg, IQR −8.3 to −5.1 and −3.9 kg, IQR −10.8 to −1.5, p = 0.028 and p = 0.005, resp.), and body fat percentage (−3.9% ± 2.8 and −1.6% ± 2.1, p = 0.010 and p = 0.036, resp.) from baseline were observed in the patients with MCGO and HSGO, respectively. In the patients with HSGO, fat-free mass decreased (−2.1 kg ± 2.2, p = 0.014) significantly as well.

#### Obesity-related comorbidities

Patients treated with liraglutide showed significant reductions in both fasting glucose levels and HbA1c. Of the patients with prior dyslipidaemia and prior elevated liver enzymes, this normalized in 1/10 (10.0%) and 3.11 (27.3%) of patients with MCGO and in 4/17 (23.5%) and 5/20 (25.0%) patients with HSGO, respectively. Specifications of the normalized parameters for all patients with resolution of prior dyslipidaemia and prior elevated liver enzymes are mentioned in Tables S5 and S6. Patients who had metabolic syndrome prior to treatment did not have metabolic syndrome anymore after treatment in 1/8 (5.6%) of patients with MCGO and 6/30 (20.0%) of patients with HSGO.

Patients treated with naltrexone-bupropion showed no significant differences from baseline in glucose indices in both groups. Of the patients with prior dyslipidaemia and prior elevated liver enzymes, this normalized in 2/7 (28.6%) and 3.6 (50.0%) of patients with MCGO and in 2/8 (25.0%) and 5/11 (45.5%) of patients with HSGO, respectively. Specifications of the normalized parameters for all patients with resolution of prior dyslipidaemia and prior elevated liver enzymes are mentioned in Tables S5 and S6. Patients who had metabolic syndrome prior to treatment did not have metabolic syndrome anymore after treatment in 1/4 (25.0%) of patients with MCGO and none of the patients with HSGO.

The analysis on the effect of weight-loss percentage on improvement of co-morbidities showed that for liraglutide 1/11 (9.1%) patient with <5% weight loss vs. 4/15 (15.4%) patients with ≥5% weight loss no longer had dyslipidaemia (p = 0.261), 5/20 (25.0%) patients with <5% weight loss vs. 3/11 (27.3%) patients with ≥5% weight loss no longer had elevated liver enzymes (p = 0.890), and 4/22 (18.2%) patients with <5% weight loss vs. 3/16 (18.8%) patients with ≥5% weight loss no longer qualified as having metabolic syndrome (p = 0.964). For naltrexone-bupropion, 2/8 (25.0%) patients with <5% weight loss vs. 2/7 (28.6%) in ≥ 5% weight loss no longer had dyslipidaemia (p = 0.876), 5/11 (45.5%) in <5% weight loss vs. 3/6 (50.0%) in ≥5% weight loss no longer had elevated liver enzymes (p = 0.858), and 0/8 (0%) patients with <5% weight loss vs. 1/5 (20.0%) patients with ≥5% weight loss resolved their metabolic syndrome (p = 0.188).

#### Quality of life

As shown in Table 3, QoL, assessed using the EQ-5D, during liraglutide treatment significantly improved in patients with HSGO (p = 0.027) and remained similar in patients with MCGO. Additionally, liraglutide treated patients with MCGO and HSGO reported a significantly improved score on the subdomain ‘Body Image’, assessed using the OBESI-Q, compared to baseline (p = 0.046 and p = 0.004, resp.).

In the group of patients treated with naltrexone-bupropion, trends towards significant improvement (p = 0.093) of QoL was observed in patients with HSGO, as assessed with EQ-5D. In addition, trends toward significant improvements were observed in patients with MCGO in the domains of ‘Eating Behaviour’, ‘Body Image’ and ‘Physical Activity’ (all p < 0.07), assessed using the OBESI-Q.

#### Side effects

71.6% of the patients treated with liraglutide and 52.4% of patients treated with naltrexone-bupropion reported gastro-intestinal side effects to some degree (Table S7). The most commonly reported side effects were nausea, constipation, diarrhoea, dyspepsia and pyrexia for both pharmacological agents. Extra gastro-intestinal side effects commonly reported were fatigue, headache and dizziness for both agents, and injection site reactions and injection site hematoma for liraglutide specifically. In the majority of patients, these side effects resolved after the dose escalation phase.

## Discussion

In this study, we show significant decreases in weight in patients with MCGO and HSGO after treatment with liraglutide or naltrexone-bupropion, in addition to conventional lifestyle treatment. Furthermore, we found significant improvements of appetite, various measures of glucose metabolism, dyslipidaemia, liver enzymes, and metabolic syndrome in both groups. Improvements in various subscales of eating behaviour and QoL were observed during liraglutide treatment mainly in patients who were highly suspected for genetic obesity. Trends toward significant improvements in several domains of quality of life were observed during naltrexone-bupropion treatment in both patients with MCGO or HSGO.

Our findings on weight loss using liraglutide and naltrexone-bupropion in patients with MCGO are in line with earlier smaller case series in patients with MC4R deficiency and 16p11.2 deletion syndrome.^16^, ^17^, ^18^ A study in 14 patients with MC4R deficiency treated with liraglutide showed a weight loss comparable to patients with non-genetic forms of obesity.^16^ Additionally, a case series of our own group (*n* = 4) on patients with MC4R deficiency and 16p11.2 deletion syndrome showed a long-term weight loss of −6.1% to −27.6% during GLP-1 analogue treatment.^17^ Recently, we published a case report demonstrating weight loss of −48.9 kg (−26.7%) after naltrexone-bupropion treatment in one patient with MC4R deficiency, who previously experienced complete weight regain after bariatric surgery.^18^ We extend the literature by demonstrating in a larger cohort that naltrexone-bupropion may also be a viable treatment option for patients highly suspected of genetic obesity. Considering the effects of the only currently available targeted therapy patients with genetic obesity, setmelanotide, liraglutide (−4.7%) and naltrexone-bupropion (−5.2%) could be considered relatively less effective compared to setmelanotide at 12 weeks of treatment (−4.2 to −16% in patients with BBS, LEPR deficiency and POMC/PCSK1 deficiency).^12^^,^^13^ We have to stress, however, that there was hardly any overlap in the genetic diagnosis included in our study and those in the setmelanotide trials. Therefore, a comparison with these trials should be made with extreme caution. Remarkably, within this cohort of therapy-resistant patients with MCGO and HSGO weight loss in response to liraglutide and naltrexone-bupropion seems comparable to the weight lost by patients with general obesity in another real-world study reporting short term data from Korea. In this study, weight loss after 4 months of liraglutide or naltrexone-bupropion treatment were −4.87% and −4.12% respectively.^32^ Other real-world studies on the effects of liraglutide on patients with common obesity have shown weight loss percentages from −4.65% to 8.7% after 4 months of treatment.^33^, ^34^, ^35^ Additionally, discontinuation of treatment due to side effects was comparable to or even less frequent in our study than in the literature for liraglutide (9.1% vs. 9.9%, resp.) and naltrexone-bupropion (16.7% vs. 24.3%, resp.).^36^^,^^37^ Our finding of weight loss in patients with MCGO and HSGO treated with naltrexone-bupropion is interesting since weight reducing properties of naltrexone-bupropion are usually attributed to dampening reward related brain structures.^38^^,^^39^ One study, however, showed that it also attenuates the hypothalamic response to food cues, suggesting an interaction between naltrexone-bupropion and hypothalamic signalling pathways such as the leptin-melanocortin pathway.^38^

The findings of appetite-improving and weight loss inducing properties of both liraglutide and naltrexone-bupropion are encouraging for patients with a clinical phenotype of a genetic obesity disorder with or without a definite diagnosis. Patients with genetic obesity disorders are known to respond less in terms of weight loss to multidisciplinary lifestyle interventions and bariatric surgery, depending on the type of genetic obesity disorder.^10^^,^^40^ Therefore, additional pharmacotherapeutical treatment options for reducing weight as well as hyperphagia, are preferred and often needed. Apart from metreleptin for leptin deficiency, the other currently available on-label and targeted pharmacotherapeutical option for genetic obesity disorders are MC4R-agonists. These are available for only a very limited number of patients with pathogenic variants in a small set of genes, including POMC, PCSK1, LEPR, and BBS, in a limited number of countries.^12^^,^^13^ Although it is currently being investigated in some of the other genetic obesity disorders, the possibility of having a wider spectrum of treatment options is desirable, e.g. in case of ineffectiveness, unavailability, or side effects of specific agents. Also, for various other genetic obesity disorders no targeted pharmaceutical option has been developed yet. In this study, we could not identify specific genotypes that consistently showed either better or poorer responses to AOM treatment compared to others. Caution is necessary due to small sample sizes. Nonetheless, the international guidelines for common obesity, including assessing treatment effectiveness after 16 weeks of treatment, could be considered for patients with genetic obesity as well.^19^^,^^20^ Especially if precision medicine is unavailable for their specific genetic condition.

Both patients treated with liraglutide and naltrexone-bupropion reported decreased appetite in a remarkable >80% in patients with MCGO as well as patients with HCGO during semi-structured interviews after 16 weeks of treatment. Interestingly, changes in eating behaviour as measured by the questionnaires we used in this study were more modest. This might suggest that current eating behaviour questionnaires do not capture the essence of hyperphagia. Recent research on setmelanotide suggests that a simple Likert Scale measured repeatedly might also be a useful parameter in measuring response in appetite.^12^^,^^13^ The definition of hyperphagia and methods to quantify hyperphagia, however, are a subject of ongoing international debate.

In patients treated with liraglutide, we observed significant improvements of both fasting glucose and HbA1c. This is probably attributed to a direct effect of liraglutide on glucose metabolism through targeting peripheral metabolic organs including skeletal muscle, next to its weight-reducing effect. Furthermore, it is encouraging that we found improvements in dyslipidaemia, elevated liver enzymes and presence of metabolic syndrome already after approximately four months of treatment across both patient groups treated with liraglutide or naltrexone-bupropion. Interestingly, in post-hoc analysis, these results seemed to be independent of the amount of weight loss. This may, however, be related to specifically the loss of fat mass. We must stress, however, that the numbers in the subgroups with these comorbidities are too small to draw strong conclusions.

During liraglutide treatment, QoL, assessed using EQ-5D, significantly improved in patients with HSGO. In naltrexone-bupropion treated patients with MCGO, we observed a trend towards improvements in QoL with respect to ‘Eating Behaviour’, ‘Body Image’ and ‘Physical Activity’, although the small number of patients limits to draw firm conclusions. These findings may be important as improving QoL is one of the treatment goals within obesity treatment.

It is essential for clinicians to have a greater awareness of the potential diagnosis of genetic obesity in adults with obesity, as this diagnosis is especially in adults often unrecognized.^41^ In numerous countries, unfortunately, access to genetic testing for these patients is limited. Together with the fact that a definitive diagnosis cannot be made in a significant number of patients despite genetic analysis, we choose to also pragmatically report the effects of anti-obesity pharmacotherapy in individuals with HSGO. Our results suggest that we can consider anti-obesity medications in patients with MCGO as well as patients with HSGO to counteract gradual progressive weight gain, and the often debilitating hyperphagia.

A strength of our study is the total sample size as this is the largest study in adults with molecularly confirmed genetic obesity treated with non-targeted anti-obesity pharmacotherapy. Furthermore, a group of patients highly suspected of genetic obesity but without definite diagnosis were included in the analyses. These patients had a similar phenotype, particularly regarding age of onset of obesity, striking weight differences with other family members, and an increased appetite. Often a definite genetic diagnosis cannot be made even though genetic testing was done or due to lack of facilities offering genetic testing. Therefore, reporting treatment outcomes in this specific group of patients with a high suspicion of genetic obesity is of paramount importance as this phenotype is probably highly prevalent across the world. A limitation of this study is that this study only describes the short-term results of pharmacological treatment of genetic obesity. We opted for treatment evaluation after 16 weeks of treatment, since the weight loss achieved in this period is useful in identifying patients who would experience further clinically meaningful weight loss, as also recommended in international guidelines.^19^, ^20^, ^21^ We do have to stress that although post-hoc power analysis showed good power for our outcomes on liraglutide and naltrexone-bupropion in the HSGO group, power was moderate in the MCGO group receiving naltrexone-bupropion. Further studies are needed to investigate the long-term results and should include preferably an even larger sample size. With many new pharmacotherapeutical options available soon, such as the long-acting GLP-1 analogue semaglutide, dual GIP/GLP-1 receptor agonist tirzepatide, triple agonist GLP-1/GIP/glucagon retratutide, and many others, new studies investigating the effects of these agents in patients with genetic obesity are needed.

In conclusion, we have shown that treatment with liraglutide or naltrexone-bupropion is associated with significant improvements in weight and appetite in a substantial portion of adults with (a clinical phenotype of) genetic obesity. We suggest that treatment with regular anti-obesity medications, in addition to conventional lifestyle treatment, can be considered in patients with a clinical phenotype of genetic obesity, while closely monitoring treatment effects and potential adverse effects. Further studies are needed to investigate the long-term effects and evaluate the effectiveness of certain anti-obesity medications for specific genetic obesity disorders or gene defects.

## Contributors

MW and CdG were involved in the conception and design of the work, the acquisition, analysis, interpretation of the data, and drafted the work. MW, CdG, EvR, and RM accessed and verified the data. MM and RM made substantial contributions to the acquisition and interpretation of the data, and critically revised the work. MB, MvH, and EvdA made substantial contributions to the interpretation of the data and critically revised the work. EvR was involved in the conception and design of the work, the acquisition, analysis, and interpretation of the data, and critically revised the work. All gave final approval for the version to be published.

## Data sharing statement

Requests for access to specific anonymised participant data can be submitted via email to the corresponding author (e.vanrossum@erasmusmc.nl). The request should be accompanied by a methodologically sound proposal on the basis of scientific merit. This proposal will be considered by the investigators, and if deemed appropriate, a data access agreement will be established after which data can be shared through a secure platform.

## Declaration of interests

The institution of MW, CdG, EvdA, and EvR has received funding for clinical trial research from Rhythm Pharmaceuticals, Inc. The current study is not linked to this study. MB and EvR receive personal royalties for the lay book FAT the secret organ. Payment to the institution or personal for scientific and/or educational presentations have been received by EvR. All other authors declare no competing interests.
