# Supplementary material for: Treatment with liraglutide or naltrexone-bupropion in patients with genetic obesity: a real-world study
Source: eClinicalMedicine. 2024 Jul 3;74:102709. doi: 10.1016/j.eclinm.2024.102709 (PMC11268126; doi:10.1016/j.eclinm.2024.102709)
Supplement: Supplementary Tables [file mmc1.docx]

**Supplementary Tables**

**Table S1. Included genetic obesity disorders**

| Affected gene | Name disease | Number of patients, n (%) |
| --- | --- | --- |
| Heterozygous MC4R |  | 8 (34.8) |
| Biallelic POMC deficiency |  | 1 (4.3) |
| 16p11.2 deletion  Distal (including SH2B1)  Proximal (excluding SH2B1) | 16p11.2 deletion syndrome | 1 (4.3)  6 (26.1) |
| Bardet Biedl genes* | Bardet Biedl syndrome | 3 (13.0) |
| *GNB1* |  | 2 (8.7) |
| *PHIP* | Chung Jansen syndrome | 1 (4.3) |
| *TRIP12* | Clark-Baraitser syndrome | 1 (4.3) |

Abbreviations: MC4R, melanocortin-4 receptor; POMC, pro-opiomelanocortin; SH2B1, SH2B Adaptor Protein 1; GNB1, G Protein Subunit Beta 1; TRIP12, Thyroid Hormone Receptor Interactor 12

* Biallelic variants were found in the following genes: BBS-1, BBS-5, and TTC-8 (resulting in BBS type 8)

**Table S2. Detected variants of unknown significance in obesity-related genes of 18 patients with HSGO**

| Affected gene | Number of patients, n (%) |
| --- | --- |
| POMC | 5 (27.8) |
| SIM1 | 4 (22.2) |
| MC4R | 2 (11.1) |
| SH2B1 | 2 (11.1) |
| LEPR | 1 (5.5) |
| PCSK1 | 1 (5.5) |
| GNAS* | 1 (5.5) |
| MC3R | 1 (5.5) |
| BBS12 | 1 (5.5) |

Abbreviations: POMC, pro-opiomelanocortin; SIM1, SIM BHLH Transcription Factor 1; MC4R, melanocortin-4 receptor; SH2B1, SH2B Adaptor Protein 1; LEPR, leptin receptor; PCSK1, Proprotein convertase subtilisin/kexin type 1; GNAS, Guanine Nucleotide binding protein, Alpha Stimulating activity polypeptide; MC3R, melanocortin-3 receptor; BBS-12, Bardet Biedl Syndrome type 12

* Variant was maternally inherited

**Table S3. Reasons for discontinuation of treatment of AOM**

|  | Liraglutide (n=84) | Naltrexone-bupropion (n=41) |
| --- | --- | --- |
| Side effects, n (%)^a^ | 5 (6.0) | 6 (14.6) |
| High costs, n (%) | 1 (1.2) | 0 |
| Due to start of other drugs not compatible with AOM, n (%) | 0 | 1 (2.4) |
| LTFU, n (%) | 0 | 1 (2.4) |

Abbreviations: AOM, anti-obesity medication; LTFU, lost to follow-up

^a^ Often due to severity of multiple side effects, which were mainly gastrointestinal of nature or fatique

**Table S4. Case descriptions of all patients who received multiple treatments consecutively**

| Patient characteristics | MCGO or HSGO | Genetic defect | AOM | Treatment outcomes |
| --- | --- | --- | --- | --- |
| Female, aged 29 years | HSGO | n.a. | 1. Liraglutide  2. Liraglutide | 1. Initial weight loss of -7.4% after 3.8 months. Liraglutide treatment was discontinued after 12 months due to an active desire to have children.  2. After 16.5 months, including a pregnancy, treatment with liraglutide was restarted. This resulted in -6.5% weight loss after 4.0 months of treatment. |
| Male, aged 40 years | MCGO | Proximal 16p11.2 deletion | 1. Liraglutide  2. Naltrexone-bupropion | 1. Initial weight loss of -4.5% and improvement of impaired appetite regulation after 4.0 months of treatment. Liraglutide treatment was discontinued after 25.9 months of treatment due to progressive weight regain and increasing appetite.  2. Two weeks later, treatment with naltrexone-bupropion was started. This resulted in +3.4% weight gain after 4.0 months of treatment. Consequently, treatment with naltrexone-bupropion was discontinued. |
| Male, aged 47 years | HSGO | Heterozygous MC4R VUS | 1. Liraglutide  2. Naltrexone-bupropion | 1. Initial weight loss of -2.9% after 4.1 months of treatment. Treatment with liraglutide was discontinued due to lack of significant response.  2. After 5.7 months, treatment with naltrexone-bupropion was started. This resulted in -5.1% weight loss after 4.8 months of treatment. |
| Male, aged 18 years | MCGO | Proximal 16p11.2 deletion | 1. Naltrexone-bupropion  2. Liraglutide  3. Naltrexone-bupropion | 1. Initial weight loss of -4.2% after 4.5 months of treatment. Treatment with naltrexone-bupropion was discontinued due to lack of significant response.  2. After 1.1 months, treatment with liraglutide was started. This resulted in +2.1% weight gain after 4.4 months of treatment.  3. As the body weight of the patient continued to increase and no other AOMs were available, treatment with naltrexone-bupropion was restarted to halt this progressive weight gain. Unfortunately, this also did not lead to weight loss (+2.1% after 5.8 months of treatment). |
| Male, aged 48 years | HSGO | n.a. | 1. Naltrexone-bupropion  2. Liraglutide  3. Liraglutide | 1. Initial weight loss of -3.1% after 5.0 months of treatment. Treatment with naltrexone-bupropion was discontinued due to lack of significant response.  2. After 1.6 months, treatment with liraglutide was started. This resulted in -7.1% weight loss after 9.0 months of treatment. This patient decided to discontinue liraglutide treatment due to the high financial burden of this treatment.  3. After a 1.4 months break, he decided to restart liraglutide treatment due to increasing weight and appetite during this break. This again led to -5.2% weight loss after 3.2 months of treatment. |
| Female, aged 47 years | HSGO |  | 1. Naltrexone-bupropion  2. Liraglutide | 1. Initial weight loss of -3.5% after 4.9 months of treatment. Treatment with naltrexone-bupropion was discontinued due to lack of significant response.  2. After 0.7 months, treatment with liraglutide was started. This resulted in -5.3% weight loss after 4.9 months of treatment. |
| Male, aged 40 years | MCGO | Biallelic BBS-5 | 1. Liraglutide  2. Naltrexone-bupropion | 1. Initial weight loss of -7.1% after 3.6 months of treatment. This patient decided to discontinue liraglutide treatment after 20.4 months due to the high financial burden of this treatment.  2. Immediately, treatment with naltrexone-bupropion was started. This resulted in -9.1% weight loss after 4.9 months of treatment. |
| Female, aged 31 years | MCGO | Heterozygous MC4R | 1. Liraglutide  2. Naltrexone-bupropion | 1. Initial weight loss of -2.5% after 5.0 months of treatment. Treatment with liraglutide was discontinued due to lack of significant response.  2. After 2.2 months, treatment with naltrexone-bupropion was started. This resulted in -15.8% weight loss after 5.9 months of treatment. |
| Female, aged 51 years | HSGO | Heterozygous POMC VUS | 1. Liraglutide  2. Naltrexone-bupropion | 1. Initial weight loss of -3.5% after 4.1 months of treatment. Treatment with liraglutide was discontinued due to lack of significant response.  2. After 4.7 months, treatment with naltrexone-bupropion was started. This resulted in -3.8% weight loss after 4.2 months of treatment. Treatment with naltrexone-bupropion was discontinued due to lack of significant response as well. |
| Female, aged 44 years | HSGO | n.a. | 1. Liraglutide  2. Naltrexone-bupropion | 1. Initial weight loss of -11.3% after 6.5 months of treatment. Treatment with liraglutide was discontinued after 18.9 months, due to because of debilitating side effects (hypoglycaemic symptoms).  2. After 5 days, treatment with naltrexone-bupropion was started. This resulted in -4.3% weight loss after 8.7 months of treatment. Treatment with naltrexone-bupropion was discontinued due to lack of significant response. |
| Female, aged 24 years | MCGO | Proximal 16p11.2 deletion | 1. Liraglutide  2. Naltrexone-bupropion | 1. Initial weight loss of -1.7% after 4.2 months of treatment. Treatment with liraglutide was discontinued due to lack of significant response.  2. After 0.7 months, treatment with naltrexone-bupropion was started. This resulted in +1.5% weight gain after 9.8 months of treatment. Even though body weight did not decrease during treatment, naltrexone-bupropion treatment was continued as she did show metabolic improvement (improved glycaemic indices, reduced dyslipidaemia parameters, and reduced waist circumference of –10 cm) and she reported an improved appetite |

Abbreviations: MCGO, molecularly confirmed genetic obesity; HSGO, patients highly suspected for genetic obesity but without definite diagnosis; n.a., not applicable; VUS, variant of unknown significance; AOM, anti-obesity medication

**Table S5. Specification of normalized parameters in patients with prior dyslipidaemia**

|  | Total cholesterol | Triglycerides | LDL-cholesterol | HDL-cholesterol |
| --- | --- | --- | --- | --- |
| Liraglutide | | | | |
| MCGO | N |  |  |  |
| HSGO | N | N | N |  |
| HSGO |  |  | N |  |
| HSGO |  | N |  |  |
| HSGO |  | N |  |  |
| Naltrexone-bupropion | | | | |
| MCGO | N |  | N |  |
| MCGO | N |  | N |  |
| HSGO | N |  | N |  |
| HSGO |  | N |  |  |

Abbreviations: LDL, low density lipoprotein; HDL, high density lipoprotein; N, normalisation

**Table S6. Specification of normalized parameters in patients with prior elevated liver enzymes**

|  | AST | ALT | GGT | AP |
| --- | --- | --- | --- | --- |
| Liraglutide | | | | |
| MCGO |  |  | N |  |
| MCGO |  |  | N |  |
| MCGO | N | N |  |  |
| HSGO | N | N |  |  |
| HSGO | N | N |  |  |
| HSGO | N | N |  |  |
| HSGO |  | N |  |  |
| HSGO |  |  |  | N |
| Naltrexone-bupropion | | | | |
| MCGO |  |  |  | N |
| MCGO |  |  |  | N |
| MCGO | N | N |  |  |
| HSGO | N | N |  |  |
| HSGO | N | N |  |  |
| HSGO |  | N |  | N |
| HSGO | N |  |  |  |
| HSGO |  |  | N |  |

Abbreviations: AST, aspartate aminotransferase; ALT, alanine aminotransferase; GGT, gamma-glutamyltransferase; AP, alkaline phosphatase; N, normalisation

**Table S7. Reported side effects during liraglutide or naltrexone-bupropion treatment**

|  | Liraglutide (*n* = 88) | Naltrexone-bupropion (*n* = 42) |
| --- | --- | --- |
| *Gastro-intestinal symptoms, n (%)* | | |
| Nausea | 46 (52.3) | 16 (38.1) |
| Constipation | 15 (17.0) | 4 (9.5) |
| Diarrhoea | 13 (14.8) | 5 (11.9) |
| Dyspepsia | 12 (13.6) | 5 (11.9) |
| Pyrosis | 10 (11.4) | 1 (2.4) |
| Vomiting | 7 (8.0) | 2 (4.8) |
| Belching | 5 (5.7) | 1 (2.4) |
| Flatulence | 2 (2.3) | 0 (0.0) |
| Changed stool pattern | 1 (1.1) | 1 (2.4) |
| Bloating | 1 (1.1) | 0 (0.0) |
| Dysgeusia | 0 (0.0) | 2 (4.8) |
| Any of above | 63 (71.6) | 22 (52.4) |
| *Other symptoms, n (%)* | | |
| Fatigue | 12 (13.6) | 8 (19.0) |
| Injection site reaction | 12 (13.6) | 0 (0.0) |
| Hematoma injection site | 8 (9.1) | 0 (0.0) |
| Headache | 7 (8.0) | 10 (23.8) |
| Dizziness | 5 (5.7) | 8 (19.0) |
| Hypoglycaemic complaints | 3 (3.4) | 0 (0.0) |
| Palpitations | 2 (2.3) | 3 (7.1) |
| Tachycardia | 2 (2.3) | 2 (4.8) |
| Increased transpiration | 2 (2.3) | 2 (4.8) |
| Mood change (e.g. feeling irritated or feeling down) | 2 (2.3) | 1 (2.4) |
| Feeling chilly | 2 (2.3) | 0 (0.0) |
| Dry mouth | 1 (1.1) | 3 (7.1) |
| Muscle ache | 1 (1.1) | 1 (2.4) |
| Hair loss | 1 (1.1) | 0 (0.0) |
| Cholelithiasis | 1 (1.1) | 0 (0.0) |
| Tremor | 0 (0.0) | 3 (7.1) |
| Feeling drowsy | 0 (0.0) | 2 (4.8) |
| Dysphagia | 0 (0.0) | 1 (2.4) |
| Allergic reaction | 0 (0.0) | 1 (2.4) |
| Polydipsia | 0 (0.0) | 1 (2.4) |
| Increased blood pressure | 0 (0.0) | 1 (2.4) |
| Tinnitus | 0 (0.0) | 1 (2.4) |
